# Measles Rash

**Published:** 1916-07

**Authors:** 


**Measles Rash** following mark of hand slap. A child was slapped by his brother just before the occurrence of measles. The rash followed the outline of the palm and finger on the cheek. ibid.

